# Will Gold Foil Cease to Be Chief of Filling-Materials?

**Published:** 1904-01

**Authors:** 


### Editorial.

### WILL GOLD FOIL CEASE TO BE CHIEF OF FILLING-MATERIALS?

The average dentist cultivated in the art of preparing cavities and filling them with gold-foil will not regard with patience any thought that would indicate an idea that gold, as a filling-material, would ever be relegated to the dust-heaps of memory. The rise of gold as a prophylactic expedient in dentistry has always been held in deep interest by those who regard its past in our work, as not merely of sentimental value, but as the foundation laid by the fathers for the upbuilding of the superstructure which we regard as our profession.

It is appropriate at the opening of a new year, in the first decade of the twentieth century, to review the past and endeavor, through observation of the traces of dental history, to read the future. The man who confines his outlook simply to the present loses that broader horizon so necessary in life's perspective. In the present number our readers will find one of the most earnest advocates of an advanced position in the use of gold-foil as a filling-material condemning the practice of looking backward, and enforces the idea that the outlook must not go beyond the present. This seems to be a narrow conception, leading to an egotistic assumption and imprisonment of mind. It is impossible to understand the present except through comparison with the past, not only in dentistry, but in all the relations of life. Experiences are too closely interwoven to admit of any isolation of periods. The human organism is to-day but the spiritual and physical unfoldment of all that has preceded it, and the dentistry of the hour is the result of the experience of all before this time.

Standing thus, as it were, between two centuries, the nineteenth and the twentieth, we may, with some degree of certainty, not only reason upon the past, but, through it, anticipate the future.

Gold-foil came into our work for the well-understood reason that it presented qualities not possessed by any other metal, and, these being recognized, it became the chief glory of dentistry that it developed processes in its use for the salvation of teeth that eventually founded a profession, one improvement leading to another, until what is known as filling teeth with gold has become a complex process, requiring not only very decided mechanical skill, but mental ability of a high order.

For decades prior to 1877 the dentists of the period regarded ability to manipulate gold-foil as the standard of excellence. The axiom prevalent, and insisted upon by the fathers, "that a tooth worth filling at all was worth filling with gold," was so impressed upon the dental mind that it required unusual courage to use any other material in permanent work. The writer, as well as the majority, was thoroughly infused with this idea, so much so, that the advocacy of the use of amalgam was regarded as a heresy not to be tolerated. The amalgam war is now part of a past history, amusing to some, instructive to others.

It was natural that this contraction of the lines of practice upon and about one material led to an effort to burst these narrow bonds, and we find a few courageous individuals secretly doing that which the ethics of the day forbid; but this made no impression, and these were regarded as unworthy of professional recognition.

There came a period, however, in 1877 when a trinity of men appeared in American dentistry, who took up the gauntlet thrown down by the fathers and asserted that, "Just in proportion as a tooth needs saving, gold is the worst material that can be used." The men who thus boldly challenged the faith of the profession were not from the ranks, but had been recognized as leaders in dental thought. This declaration jarred dentistry in America from centre to circumference, and the "New Departure," as it was called, was denounced upon every side as the creation of a set of iconoclasts who delighted in tearing down our golden images and giving nothing in their place worthy of acceptance. The names of Flagg, Palmer, and Chase were not, therefore, regarded in an enviable light; indeed, their names and work became a byword and reproach with the unthinking crowd.

It is rare that contemporaneous thought rises to a full conception of advanced ideas. The mind is narrowed by environment, and time alone can raise the mist to a clearer conception of the possible effect of things, new and strange, upon the work of individuals and the world. Thus it was that the ideas of the New Departure advocates fell, apparently, for the time unheeded.

It was only apparent, however, for it was soon evident that the thinking portion of the profession were beginning to ask themselves the question, Have we not been too long worshipping a golden fetish, that has not only been to some extent an injury to our patients, but also to our own loss? It did not take long for the answer to come, and this was demonstrated in an intelligent revision of the entire subject. Amalgam began to be used by the best gold operators where time, money, and strength had been exhausted previously in large gold operations. Plastics of all kinds were adopted where indicated, as the most satisfactory material for the case in charge, and while gold still maintained its preeminence, and the undergraduate was still taught to reverence it as chief of materials in the salvation of teeth, it has measurably fallen from its high estate, and the old axiom is no longer an ethical test to be used by the leaders of dental thought.

If we may judge from the past, taking in view the origin of gold as a filling-material, until the period when it reached its greatest perfection in practice to its decline as an absolute standard of excellence, we may confidently assume that the day will come when its use will be classed as belonging to the barbaric period in dentistry. This may seem an impossible conception, but we have not far to go for a basis upon which to build a prophecy of the future. It is very possible that the present craze for porcelain fillings may eventually reach a lower level than it occupies to-day, but the present indications point directly to the time when it will supplant gold in the large majority of cases coming under dental care, and that plastics, in some form, will subserve dental purposes for the remainder. This, to the gold worshipper, is an inconceivable idea, but if he will only review his dental history he will find, as stated, that there has been a gradual but very sure departure from the old ideas, and dentistry, in this country, could not go back, even if desirable so to do, to the period antedating the new departure creed. Change is in the air and the restless life, ever seeking something new, will drive us on until gold is no longer known as a filling-material for the salvation of teeth.

It seems appropriate that this matter should be discussed at some length, in view of the recent departure of the earnest leader of the three men who made progress in this direction possible.

Dr. J. Foster Flagg was regarded as an extremist, and it is very true that he was a radical of radicals in his views upon this subject; but while he stood apart among his fellows, it required just such self-abnegation, just that devotion to an idea, which he exemplified, to make an impression. A weak man would have failed utterly. He, with his colleagues, forced thought, and with thought came action. We may not regard his postulate as being strictly true, but that is unnecessary, for all truth cannot be formulated in a sentence, neither is an oak-tree found entire in the acorn. It is the aggregation of various influences that create revelations. They are not all fully developed at the moment of inspiration.

The writer does not wish to be misunderstood in this connection. He is fully in accord with those who hold to the highest development of gold as a filling-material. It, at present, constitutes that which has stood the test of time, but he recognizes the fact that it is, at its best, but a temporary expedient. What he desires is to enforce the truth, made prominent by the new departure advocates, that age, temperament, quality of tooth-material, and constitutional conditions must all be considered in deciding the question of what is best to use to save teeth. Dr. Flagg answered this when he wrote, in 1888, "For teeth of good structure and with cavities in accessible positions, gold is the king of filling-materials."

If we cannot agree entirely with the iconoclasts of 1877, let us, at least, honor them as earnest men who blazed a path in which the profession must tread reverently. The New Departure has become even now the old departure. The men who originated it have passed to other activities, but the influence they generated will grow and the materials in use in the future will be more in harmony with the structure of which nature has formed the teeth of the human race, and when that time comes the dentist of the period will truly have cause to remember the names of Flagg, Palmer, and Chase as the men who trained the thought from a close adherence to gold-foil to a more reasonable conception of what is needed for the true progress of dentistry and the salvation of all classes of teeth.

